# Inflammation and cardiac dysfunction during sepsis, muscular dystrophy, and myocarditis

**DOI:** 10.4103/2321-3868.123072

**Published:** 2013-12-18

**Authors:** Ying Li, Shuping Ge, Yizhi Peng, Xiongwen Chen

**Affiliations:** 1 Institute of Burn Research, Southwest Hospital, State Key Laboratory of Trauma, Burns and Combined Injury, the Third Military Medical University, Chongqing, 400038 China; 2 Cardiovascular Research Center and Department of Physiology, Temple University School of Medicine, Philadelphia, Pennsylvania 19040 USA; 3 Drexel University College of Medicine, Philadelphia, Pennsylvania USA

**Keywords:** Burn, inflammation, sepsis, Duchenne muscular dystrophy, cardiac dysfunction, contractility

## Abstract

Inflammation plays an important role in cardiac dysfunction under different situations. Acute systemic inflammation occurring in patients with severe burns, trauma, and inflammatory diseases causes cardiac dysfunction, which is one of the leading causes of mortality in these patients. Acute sepsis decreases cardiac contractility and impairs myocardial compliance. Chronic inflammation such as that occurring in Duchenne muscular dystropshy and myocarditis may cause adverse cardiac remodeling including myocyte hypertrophy and death, fibrosis, and altered myocyte function. However, the underlying cellular and molecular mechanisms for inflammatory cardiomyopathy are still controversial probably due to multiple factors involved. Potential mechanisms include the change in circulating blood volume; a direct inhibition of myocyte contractility by cytokines (tumor necrosis factor (TNF)-α, interleukin (IL)-1β); abnormal nitric oxide and reactive oxygen species (ROS) signaling; mitochondrial dysfunction; abnormal excitation-contraction coupling; and reduced calcium sensitivity at the myofibrillar level and blunted β-adrenergic signaling. This review will summarize recent advances in diagnostic technology, mechanisms, and potential therapeutic strategies for inflammation-induced cardiac dysfunction.

## Introduction

Inflammation is a defending biological process to fence off injurious stimuli such as pathogens, damaged tissues, or irritants. Temporally it can occur acutely or chronically. In term of the scale of inflammation, it can occur locally confined within local tissues/organs or systemically (whole body). Acute and/or systemic inflammation often accompanies with a lot of diseases. Systemic inflammatory response always has noninfectious causes such as trauma and burns or infectious causes such as viral or bacterial infection. Though appropriate extent of inflammation is protective and beneficial to maintain homeostasis of the organism, excessive and chronic inflammation is detrimental in that it alters many vital organs. As we know, burn injury can lead to global immunological changes including suppressed immune function and increased susceptibility to infection.[1] Sepsis and septic shock are common complications in severe burn patients, and lead to high morbidity and mortality in the burn intensive care units.[2,3] In response to burn-induced sepsis, cardiomyocytes release proinflammatory cytokines, such as tumor necrosis factor (TNF)-α, interleukin (IL)-1β, IL-6, and nitric oxide (NO) to initiate a local inflammatory response.[4] At the cell level, local or circulating inflammatory factors decrease cardiac myocyte contractility directly and affect the overall repair process. And in some chronic inflammation disease, such as diabetes, atherosclerosis, heart failure, and muscular dystrophy, the inflammatory response acts both as the reason and the result of these chronic diseases and is a critical factor for the clinical outcomes.[5] Inflammation, especially sepsis (a severe form of systemic inflammation), causes both impaired systolic function (cardiac depression) and diastolic function. Echocardiography and hemodynamic measurements often show reduced ejection fraction, abnormal tissue motion, and relaxation impairment. Electrical disturbances (arrhythmias) are often observed in these patients as well. Serum biomarkers for cardiac dysfunction such as troponin T, troponin I, and brain natriuretic factor (BNF) are often increased.[6,7] This review will summarize the clinical presentation and potential mechanisms of acute and chronic inflammation induced cardiac contractility depression, and further address the advanced therapies to support cardiac recovery in sepsis-induced cardiac dysfunction patients.

**Table 1:** 

Access this article online	
**Quick Response Code:**	**Website:** www.burnstrauma.com
	**DOI:** 10.4103/2321-3868.123072

## Inflammation

Inflammation is an active biological process involving multiple components to clean pathogens (e.g., viruses, bacteria, parasites, or other invading particles such as allergens), injured tissue/cells (e.g., necrotic cells), or to respond to abnormal systemic function such as autoimmunity and lipoprotein deposition.[8] It intends to remove harmful invading pathogens or injured tissue or undesired molecules to restore tissue homeostasis and function. Inflammation can be divided into different forms according to the time frame of inflammation development and resolution, the mechanisms of induction, regulation, and resolution. Local acute inflammation is often manifested with redness, swelling, heat, and pain. Another major sign of inflammation is the disturbance of tissue or organ function.[9] However, uncontrolled chronic inflammation is the cause for many diseases like diabetes, atherosclerosis, degenerative neurological diseases (e.g., Alzheimer's disease), and cancer.[10] The inflammation process usually involves four components: the inducers, the sensors, the inflammatory mediators, and the target tissues. Inducers including pathogens, injured tissues, and other abnormal components in the body (e.g., monosodium urate crystals) can be sensed by pattern recognition receptors (PRRs), which are represented by Toll-like receptors (TLRs) and NOD-like receptors.[11] Subsequently, transcription factors like nuclear factor κB (NF-κB) and activating protein 1 (AP-1) are activated in immune cells to further induce the production of cytokines, chemokines, and antimicrobial factors.[12] As such, the inflammatory response is initiated and maintained till the stage of “the resolution of inflammation” when pathogens or injured tissues are cleared. At this stage, the proinflammatory signals are switched to signals promoting tissue repair. However, for many reasons, the inducers (e.g., autoimmunity[13] and deposited lipoproteins) for inflammation cannot be cleared in a short period. Persistent presence of inducers and positive reinforcement between the inducers and inflammation response result in chronic inflammation, contributing to the development of many chronic inflammatory diseases such as rheumatoid arthritis, type 2 diabetes, degenerative neurological diseases, and cancer.[14] Therefore, anti-inflammation has been considered effective therapeutics for many of these diseases.[13]

Both acute and chronic inflammation has local or systemic response. In some severe infectious diseases, especially when the bacteria or pathogen were expanded in the vascular system, systemic inflammation response occurs. When activated immune cells, cytokines/chemokines, and tissue debris during acute inflammation are produced to such a scale that they are present in the circulation at high concentrations, acute inflammation becomes systemic and septic. Chronic inflammation happening in the vasculature, the fat tissue of obesity, and skeletal muscles of muscular dystrophy is also systemic. Local inflammation in vital organs such as the heart in ischemia/reperfusion injury impairs the function of the organ; systemic inflammation in sepsis may depress organ function remote from the inflammatory sites.

## Normal cardiac function and regulation

The heart works as a pump to circulate the blood through the whole body, carrying necessary nutrients and oxygen to the peripheral tissues and removing metabolic waste and CO_2_ from the tissues. Cardiac function is constantly regulated by the sympathetic and parasympathetic nervous systems (SNS and PSNS) to meet different needs of the body under different physiological and pathological conditions.[15,16] When the adrenergic/SNS is activated, catecholamines are released to activate β-adrenergic receptors/stimulatory G protein (Gs)/adenylyl cyclase (AC)/cyclic adenosine monophosphate (cAMP) signaling pathway in cardiac myocytes, inducing chronotropic (increasing the heart rate), iontropic (increasing cardiac contractility), and lusitropic (increasing the rate of cardiac relaxation) effects. Elevated cAMP in the sinoatrial node (SAN) cells directly increases the funny current (I_f_) and activates protein kinase A (PKA) to enhance Ca^2+^ handling to accelerate the automaticity of these cells and the heart rate (chronotropy).[17] The pump function of the heart depends on cyclic contraction and relaxation of the contractile heart cells (cardiac myocytes) in concert.

### Calcium handling and excitation-contraction coupling in normal cardiac myocytes

Calcium (Ca^2+^) plays a critical role in the contraction of muscles including skeletal, cardiac, and smooth muscles. Rhythmic rise and decay of cytosolic Ca^2+^ (calcium transient) activates myocyte contraction. The rhythm of calcium transients is dictated by the excitation-contraction coupling process: When an electrical pulse propagates from the SAN to the atria and ventricles, it causes depolarization of cardiac myocytes by opening Na^+^ channels. The depolarization of cardiac myocytes activates the L-type Ca^2+^ channels (LTCC) causing the opening of these channels. The small Ca^2+^ influx through the LTCC causes the release of Ca^2+^ from the sarcoplasmic reticulum (SR) through the ryanodine receptor (RyR) via a Ca^2+^-induced Ca^2+^ release mechanism (CICR).[18,19] The binding of Ca^2+^ to troponin C removes the inhibitory effect of troponin I on the interaction between thin myofilament (actin) and thick myofilament (myosin) and initiates myocyte contraction. Subsequently, actin activates the myosin Ca^2+^-adenosine triphosphatase (ATPase) which hydrolyzes adenosine triphosphate (ATP) to cause the sliding of cross-bridges between the thin (actin) and thick filaments (myosin) and the cell develops force and shortens. This process is termed “excitation-contraction coupling” (EC coupling).[20] Cytosolic Ca^2+^ is then removed by three pathways: Sequestrated back into the SR via the sarco/endoplasmic reticulum Ca^2+^-ATPase (SERCA), pumped out of the cell via sarcolemmal Ca^2+^-ATPase and transported out of the cell by the Na^+^/Ca^2+^ exchange (NCX). As the cytosolic free Ca^2+^ concentration decays, the myocyte relaxes.

**Figure 1:**
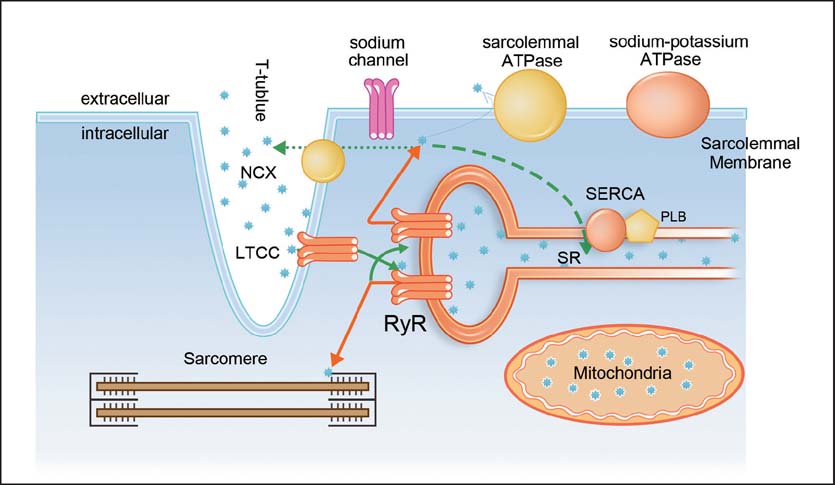
Scheme of cellular structures and molecules involved in excitation-contraction coupling in ventricular myocytes. The black stars indicate Ca^2+^ ions. Solid lines indicate Ca^2+^ influx into cytosol and dotted lines indicate Ca^2+^ extrusion pathways. The thickness of the line indicates the relative contribution of each pathway. LTCC = L-type Ca^2+^ channel, NCX = sodium-calcium exchanger, PLB = phospholamban, RyR = ryanodine receptor, SR = sarcoplasmic reticulum, SERCA = sarcoplasmic reticulum Ca^2+^ ATPase.

This CICR process is facilitated by the special cardiac myocyte structure: The sarcolemmal membrane perpendicularly invaginates near the Z band where thin myofilaments attach and forms a specific subcellular structure termed “transverse (t) tubule system”. The sarcolemmal membrane of the t-tubules and the membrane of the SR in close apposition (junctional SR membrane) form the structure dyad. The critical molecules involved in CICR, the LTCC and the RyR, are mainly located at the t-tubules and the junctional SR membrane, respectively [Figure 1]. The cleft between the membrane of the junctional SR and the membrane of the t-tubule is very narrow (∼12 nm). On the longitudinal SR membrane, there are phospholamban (PLB) and SERCA, which plays a major role in resequestrating Ca^2+^ back into the SR. The NCX is preferentially distributed within the t-tubules or on the membrane close to the t-tubules and sarcolemmal Ca^2+^-ATPase is evenly distributed on the sarcolemmal membrane.[21]

### The regulation of excitation-contraction coupling (EC) coupling and cardiac contractility by the sympathetic system

As aforementioned, cardiac contractility is mainly enhanced by the SNS. In cardiac myocytes, cyclic AMP increased by released catecholamines from the SNS activates PKA by binding to the regulatory subunit of PKA (PKA R) and relieving its inhibitory effect on the catalytic subunit of PKA (PKA C). PKA phosphorylates the LTCC (the responsible phosphorylation sites still controversial), PLB (at Ser16) and RyR2 (at Ser2814) to increase the activities of the LTCC, SERCA and RyR2. Consequently, the phosphorylation of these molecules by PKA results into a larger Ca^2+^ influx to load the SR, and a greater SR Ca^2+^ content, a stronger trigger for excitation-contraction coupling (ECC) and a faster, more coordinated and larger Ca^2+^ release from the SR. The greater Ca^2+^ release into the cytosol (a greater Ca^2+^ transient) induces stronger myocyte and cardiac contraction (inotropy). PKA also phosphorylates troponin I, reducing Ca^2+^ affinity of the myofilament and thus promotes the dissociation of Ca^2+^ from the myofilament. This effect of PKA together with enhanced SERCA activity makes the removal of Ca^2+^ from the cytosol and the relaxation of the cardiac muscle faster and produces the lusitropic effect (lusitropy).[22] Recently, another cAMP sensor, the exchange protein directly activated by cAMP (EPAC), has been proposed to mediate the positive inotropic effect of the SNS excitation via phospholipase C and Ca^2+^/calmodulin-dependent protein kinase II (CaMKII).[23–27] However, the role of EPAC in acute regulation of Ca^2+^ handling in cardiac myocytes is still controversial.[28]

## Cardiac dysfunction during inflammation

### Diagnostic technology of monitoring cardiac function

With invasive hemodynamic measurements, it was decades ago several animal studies and human observations have demonstrated impaired left ventricular systolic and diastolic function in septic shock.[29,30] Researchers also found abnormalities and parallel alterations in the right ventricle.[31] Nowadays, with the development of advanced diagnostic technology, the evidence of depressed myocardial function in sepsis patients exists not only in the abnormal hemodynamics, but also in some other parameters. Transthoracic echocardiography has become an important diagnostic tool for continuously monitoring cardiac function during hospitalization and provides real-time and direct information on cardiac performance rather than indirect implications of clinical signs alone; radionuclide techniques can also provide many of these parameters but the additional risk of radiation exposure limits its application in the critically ill patients. Doppler techniques are noninvasive and simple to operate, but limited data can be obtained from this technique alone. Pulmonary artery catheter has been used for monitoring of myocardial performance in the critically ill over 20 years; however, how it should influence therapeutic decisions in clinic is still controversial.

### Cardiac dysfunction in acute inflammation/sepsis

Sepsis has been characterized as an acute and complex systemic immune and inflammatory response to infections.[32] It usually occurs after acute and systemic injury, such as severe burn, trauma, and surgery, and leads to severe sepsis with acute organ dysfunction and septic shock (severe sepsis with persistent hypotension and not reversed with fluid resuscitation) when the host response to the global inflammatory response were amplified and dysregulated.[33] Myocardial dysfunction is present in 40% of sepsis patients and the mortality of sepsis patients with cardiac dysfunction is up to 70%. Although sepsis-induced myocardial suppression is occasionally lethal, moderate sepsis induces cardiac “hibernation” that could be advantageous by preventing stress induced cardiac injury.[34–36] However, to date the mechanistic basis for sepsis-induced cardiac dysfunction is still controversial.[6,7]

In the clinic, there are two types of sepsis-associated cardiovascular dysfunction according to the distinct hemodynamic characteristic: One is hyperdynamic state also called warm shock and the other is hypodynamic state also called cold shock. Warm shock was characterized by normal or high cardiac output and low systemic vascular resistance,[37] with which patients always have warm and flushed skin. Cold shock is associated with decreased cardiac output, hypotension, and clammy skin. Cold shock is more common and much more severe than warm shock. In the early stage of sepsis, decreased global blood volume leads to low cardiac output. With aggressive fluid resuscitation and improvement in intravascular volume, and in combination of septic shock patients’ low systemic vascular resistance, many patients develop a high cardiac output.

### Cardiac dysfunction in chronic inflammation caused by muscular dystrophy

Chronic inflammation occurring systemically or locally in the heart also has significant impact on cardiac function through many mechanisms. As the underlying causes of inflammation in chronic inflammation are different from those of acute inflammation, the underlying mechanisms for cardiac dysfunction by chronic inflammation include some distinct signaling pathways. We will use cardiac dysfunction induced by the chronic inflammation condition occurring in muscular dystrophy as an example here.

Duchenne muscular dystrophy (DMD) is a rare inherited, X-linked recessive disease affecting the function of skeletal and cardiac muscles in 1 out of 3,600–6,000 live newborn males.[38] DMD patients have very poor quality of life and prognosis.[38] It leads to muscular dystrophy and eventually death in the young males. The early manifestation of this disease is the weakness in skeletal muscles and the loss of mobility due to the degeneration of skeletal muscles.[39] Weakening of the diaphragm results in the death of DMD patient due to respiratory failure. However, now with ventilation support, DMD patients can survive longer than a decade ago.[39] Thus more DMD patients are surviving with cardiac dysfunction and eventually dilated cardiomyopathy.[39] Cardiac dysfunction at rest is detected at the age of early teens or even at the age of 8–9 years with three-dimensional (3D) strain, torsion, and dispersion analyses.[40,44]

Cardiac dysfunction in DMD has long been recognized with initial pathology including myocyte hypertrophy, myocardial fibrosis, typical electrocardiographic abnormalities, and abnormal wall motion detected by early echocardiography. Cardiomyopathy in DMD generally starts as a preclinical or intermediate stage, with symptoms and signs of heart failure such as dyspnea, peripheral edema, and liver enlargement. However, in few patients the dilation could be the first manifestation of the heart involvement, caused by a diffusing disorganized fibrosis. The ability to detect overt cardiomyopathy increases with age, so that more than 80% of boys older than 18 years will have abnormal systolic function.[45]

Though it is believed that cardiac myocyte death is due to the loss of membrane integrity, Ca^2+^ overload and NO over production is the major contributor to cardiac dysfunction in DMD patients,[46,47] the underlying mechanisms for the development of dilated cardiomyopathy in DMD patients is still not entirely clear. Massive death of myocytes in the skeletal and cardiac muscles results in intense inflammation[48] and prostaglandin E2 (PGE2) production in DMD patients.[49,50] The role of systemic inflammation and local cardiac inflammation in cardiac dysfunction in DMD is still unclear. As we have recently shown, PGE2 is able to inhibit acute α-adrenergic response via a phosphodiesterase-coordinated local cAMP regulation.[51] We have also observed that there is a blunted cardiac α-adrenergic response of the heart of the mdx mouse, a mouse model of human DMD.

### Cardiac dysfunction with initial acute inflammation followed by chronic inflammation

Some events such as myocarditis lead to acute inflammation then followed by chronic inflammation. Myocarditis is an inflammation of cardiac muscle caused by infectious (bacterial, viral, and fungal) and noninfectious (autoimmune, toxic, drug-induced hypersensitive, and vasculitic) insults that first induce an acute inflammation, and then chronic inflammation if the pathogens cannot be completely removed.[52] The incidence of myocarditis is not exactly known, but it accounts for 9.6% of unexplained heart failure and 12% of sudden death of patients younger than 40.[53] Approximately 21% patients having acute myocarditis develop dilated myopathy.[54] The 5-year survival rate of biopsy-proven myocarditis is about 50%. Virus infection is the most often cause of myocarditis.[52] During past 20 years, there is a shift of the virus causing myocarditis: In the middle and late 1990s, enterovirus especially Coxsackie B virus, was the main cause of viral myocarditis; then adenoviruses were found as the major virus; in recent years, parvovirus B19 was found to be the major virus.[52] Myocarditis can be divided into three phases: Initial viral infection activating innate immune response by lysing cardiomyocytes and activating antigen-presenting cells; usually viruses are cleaned during the first phase without much damage to the heart. However, if viruses are not cleaned, the second phase with adaptive immune response is ensued. The adaptive immune response is more specific with T-cells and antibodies against some cardiac proteins such as myosin and α-adrenergic receptor. In most patients, the viruses are cleaned after that but few patients may advance into the chronic “inflammatory cardiomyopathy” phase, which causes diastolic cardiomyopathy with depressed cardiac function.[52]

Most myocarditis patients recover after the initial phase without any clinical cardiac dysfunction. However, in some patients with myocarditis, acute inflammation could cause acute heart failure, arrhythmias, and sudden cardiac death.[52,55] A study by the European Study of the Epidemiology and Treatment of Cardiac Inflammatory Disease (ESETCID) found that 72% of myocarditis patients had dyspnea, 32% had chest pain and 18% had arrhythmias.[55] Physical examinations could find tachycardia, soft S1 sounds and S3 and S4 gallop, nonspecific ST-T wave changes, atrioventricular block, and intraventricular block.[56] In some patients, there are acute infarction like symptoms.

## Mechanisms for cardiac dysfunction induced by inflammation

### Mechanisms for cardiac dysfunction during sepsis

Reversible myocardial depression is common in human septic shock. In most septic shock patients, ventricular dysfunction was evident at admission. Hemodynamic or echocardiography examination always show ventricular dilatation and decreased left ventricular ejection fraction (LVEF), and these abnormalities were more pronounced in survivors than in nonsurvivors. At the same time, survivors have better response with hemodynamic support during the monitoring period.[57] However, the latest metaanalysis did not show lower ejection fractions in survivors than nonsurvivors of severe sepsis or septic shock.[58] Early understanding of cardiac dysfunction in sepsis was based on the hypothesis of global myocardial ischemia, but later studies in animal and human showed high coronary blood flow and decreased myocardial oxygen utilization in sepsis.[59] Furthermore, studies found no significant changes at the organ, cellular, and molecular levels of depressed hearts of septic patients. Takasu *et al.*, found myocyte death is rare in sepsis induced cardiac dysfunction patients.[60] All the evidence supports that sepsis-induced myocardial dysfunction is an acute and reversible response and much more like a self-protection response in emergency, so functional changes rather than anatomical abnormalities is the underlying pathophysiology. However, cardiac dysfunction in sepsis is multifactorial and involves multiple and complex pathways.

**Figure 2:**
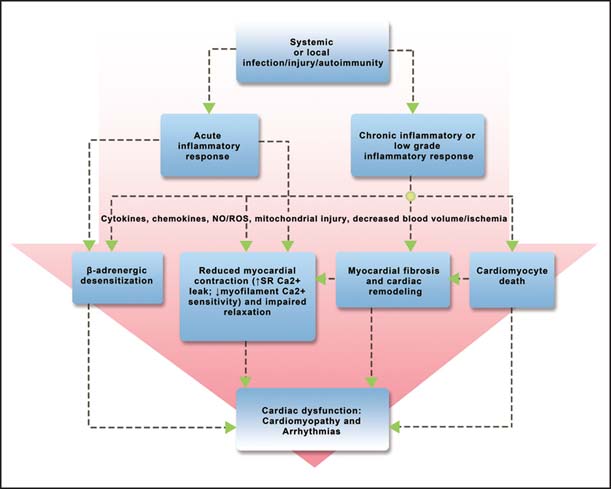
Mechanisms for cardiac dysfunction induced by inflammation.

As mentioned above, most evidence showed acute cardiac dysfunction induced by septic stress is reversible in survivors within 7–10 days, supporting that the pathophysiology basi| is functional rather than structural. The mechanisms of myocardial dysfunction are related to inflammatory response mediated by cytokines, oxidative stress and mitochondrial abnormalities, alterations of micro- and macrocirculation, metabolic changes and autonomic dysfunction, and NO and a derangement in catecholaminergic stimulation.[7,61] Endothelial dysfunction with consequent microcirculatory and mitochondrial dysfunction, and circulating factors are considered to be the most important mechanisms of acute inflammation induced myocardial dysfunction[62] [Figure 2].

Cytokines act as the most important mediators of the inflammatory process. TNF-α and IL-1β are postulated to play a key pathophysiologic role during sepsis. Serum levels of TNF-α and IL-1β are increased during an inflammatory response, imposing a negative inotropic effect on cardiomyocyte contractility.[63,64] Circulating IL-6, IL-8, and IL-10 are potent cardiomyocyte contractility depressor.[65] Some *in vitro* studies have shown that the depression of cardiomyocyte contractility induced by septic serum is not directly dependent on elevated levels of TNF-α and IL-1β, but Duncan *et al.* revealed that TNF-α and 1L-1β increase the SR Ca^2+^ leak from the SR, contributing to the depressed Ca^2+^ transient and contractility.[66] Maass and coworkers found that burn injury or burn serum pretreatments increases cardiomyocyte cytosolic and mitochondrial Ca^2+^ and promotes myocyte secretion of TNF-α, IL-1β, and IL-6, which induce mitochondrial injury of cardiomyocytes during sepsis and burn trauma.[67] The circulating levels of thrombopoietin (TPO) is increased by as much as two-folds than in the healthy person accompanied by increased monocyte-platelet aggregation (i.e., P-selectin expression) in burn patients with sepsis.[68] Much has been done to verify the important role of TLR4 as a mediator of septic shock and myocardial infarction (MI)-induced cardiac dysfunction in the acute phase as well.[4] Besides classical cytokines, some new inflammation mediators (IL-7, IL-17A, IL-22, and IL-33), soluble receptor sTREM-1, stress mediators HMGB1, histones glycoprotein osteoponitin, lipid mediators (S1P and RvD2), resistin adipokines (adiponectin and visfatin), vasoactive peptides (ghrelin, AM/AMBP-1, and ET-1), and growth factor (MFC-E8) were verified to participate in the inflammatory response during sepsis.[69] However, the roles of these mediators in cardiac dysfunction during sepsis have not been studied.

NO not only plays an important role in the development of sepsis induced cardiac dysfunction, but also has protective effects. Chronic stress and inflammation have dysfunctional NO signaling and insulin resistance which affect many tissues, including the vasculature, the myocardium, and the musculature.[70] The ensuing vascular dysfunction and metabolic disturbances over time evolve into cardiometabolic diseases.[70] The high level of NO produced by NO synthase 2 (NOS_2_) leads to systemic hypotension and myocardial dysfunction associated with sepsis. The increases in NO production during sepsis can increase S-nitrosylation of proteins that may lead to cardiac dysfunction. Sips *et al.*, found that increasing S-nitrosoglutathione reductase (GSNOR), an enzyme promoting denitrosylation activity, can improve myocardial dysfunction during sepsis by reducing protein S-nitrosylation during sepsis and thus increasing cardiac myofilament sensitivity to Ca^2+^.[71] However, clinical trials using nonselective NOS inhibitors showed increased mortality in septic patients, suggesting a protective role of nitric oxide synthase 1 (neuronal NOS) (NOS1) and/or nitric oxide synthase 3 (endothelial NOS) (NOS3) in sepsis.[72] Cardiomyocyte-specific NOS3 overexpression mice having increased myocardial NO levels can attenuate endotoxin-induced reactive oxygen species (ROS) production and increase total SR Ca^2+^ load and myofilament sensitivity to Ca^2+^, thereby reducing cardiac depression (reduced cardiac contractility) in septic shock mice.[72] It seems that local NO production combined with cytokine release plays a key pathophysiological role during early stage of sepsis.[73]

Mitochondrial derangement plays a key role in the mitochondrial bioenergetic dysfunction in tissue injury and sepsis-associated multiorgan failure.[74] Cell death is rare in sepsis-induced cardiac dysfunction, but sepsis-induced focal mitochondrial injury occurs. Though till now there is no direct evidence to prove the relationship between the morphologic change of mitochondria and cardiomyocyte function depression, the mitochondrial swelling of the septic cardiomyocyte is relevant to sepsis-induced myocardial depression.[60] Zang *et al.*, demonstrated that sepsis leads to mitochondria membrane damage to increase ROS and change the defense capability to ROS;[75] they also found that inhibiting of mitochondrial ROS by a mitochondria-targeted vitamin E in a sepsis animal model can protect mitochondrial function and attenuate tissue-level inflammation to improve cardiac function during sepsis.[76]

Altered myofilament Ca^2+^ sensitivity, abnormal calcium homeostasis, and defects in cardiomyocyte coupling by gap junctions have also been proposed as potential causes of sepsis-induced cardiac depression though it is still unclear which is the major cause. Many studies have shown reduced Ca^2+^ sensitivity of the myofilament of cardiomyocytes during sepsis.[77,78] The reduced myofilament Ca^2+^ sensitivity is more related to the changes of the regulatory proteins (tropomyosin and troponin) rather than the changes of the structural myofilament proteins (actin and myosin) because the maximal Ca^2+^-activated tension tends to be unchanged.[77,79] Levosimendan, a troponin-C Ca^2+^-sensitizer, markedly enhanced left ventricular function in animals with experimental septic shock,[80] but Behrends and Peters[81] reported reduced Ca^2+^ sensitivity during sepsis was not due to troponin-C, but probably increased troponin-I phosphorylation.[79] There are also reports showing that critical molecules involved in Ca^2+^ handling including the L-type Ca^2+^ channel,[82] the Ca^2+^ release channel RyR2[83] and SERCA,[84] are reduced during sepsis, contributing to reduced cardiac myocyte contractility. Also, the phosphorylation of phospholamban could be decreased, enhancing the inhibitory effect of PLB on SERCA and reducing Ca^2+^ uptake into the SR.[85] The potential increased Ca^2+^ leak from the SR through RyR may decrease SR Ca^2+^ content and increase diastolic Ca^2+^ to cause both systolic and diastolic cardiac dysfunction.[86] However, the molecular signaling involved in inflammation-regulated Ca^2+^ handling needs to be further defined.

An underlying mechanism for cardiac depression after acute or chronic inflammation is that excess activation of the SNS leads to adverse cardiac remodeling and a blunted adrenergic response. But how the cytokines or inflammation signal affects β-adrenergic signal is not clear. PGE2 is found to be elevated quickly after burn or in sepsis.[87] We recently found that PGE2 stimulation can attenuate the adrenergic-induced cardiac contractile response in animal hearts and cardiac myocytes via activating phosphodiesterase 4Ds (PDE4Ds), a novel mechanism might contribute to reduced β-adrenergic reserve of the heart,[51] but also provide cardioprotection against β-adrenergic overactivation.

During chronic inflammation, the cytokines, chemokine, NO, ROS, and altered Ca^2+^ handling could have similar effects as they exert during acute inflammation but in general, local concentrations of these factors may be low. However, prolonged presence of these factors at low concentrations induces adverse cardiac remodeling via promoting myocyte hypertrophy and apoptosis, the differentiation of fibroblast into myofibroblasts and extracellular matrix protein production (fibrosis).[88] Myocardium fibrosis impairs cardiac diastolic function. Both Ca^2+^ and NO in cardiac and respiratory muscle pathways have been shown to be important to the etiology of DMD. In DMD, the preservation of relatively normal calcium reuptake and diastolic/systolic sheet mechanics throughout the rest of the heart, together with the rapid reversibility of functional defects by reducing cytosolic calcium, points to the significance of regional mechanical factors in the progression of the disease.[89] Interestingly, some inflammatory factors in chronic inflammation can be both detrimental and beneficial. For example, while TLR4 initiates inflammation, it might also protect the myocardium.[90] The activation of TLRs leads to nuclear translocation of NF-κB to induce the production of cytokines and adhesion molecules in immune cells and nonimmune cells such as endothelial cells, fibroblasts, and cardiomyocytes. These cytokines plays critical roles in myocarditis and cardiomyopathy.[91] Protease activated receptor 1 and2 (PAR1 and PAR2),[92] IL-1β,[93]IL-6,[94] and IL-18[95] also stimulate cardiomyocyte hypertrophy. IL-18 may be proapoptotic and prohypertrophic as well.[95]

In summary, the mechanisms for cardiac dysfunction induced by inflammation have not been fully understood and it seems that multiple underlying mechanisms for cardiac dysfunction for both acute and chronic inflammation are overlapping. There could be difference in the relative contribution to each inflammatory condition and to different stages of inflammation.

## Treatments

### General considerations for treating cardiac dysfunction during inflammation

The guidelines for management of severe sepsis and septic shock[33] provide details about the grading and evaluation system, and different treatments for different stage of sepsis and septic shock. The guidelines also emphasize the importance that acute management of sepsis and septic shock is the foundation of improved outcomes for this group of critically ill patients. The basic principle for treating diseases is early diagnosis for early treatment, so is the same for sepsis induced myocardial dysfunction. Early detection of sepsis induced myocardial injury is based on the biomarkers and noninvasive diagnostic methods. Cardiac troponins are regulatory proteins of the cardiac muscle, and they are highly sensitive and specific markers of myocardial damage. Cardiac troponin I (cTnI) and cardiac troponin T (cTnT) are released as a result of myocardial cell injury though they are also elevated in some other acute diseases, such as acute coronary syndrome, acute kidney injury, and pulmonary embolism. High troponin or brain natriuretic peptide (BNP) levels are associated with myocardial dysfunction and a higher mortality.[96] In Lupia *et al.*’s study, they found increased circulating levels of TPO after burn injury, as much as two-fold of that in the healthy. Additionally, they also found increased monocyte platelet aggregation in burn patients with sepsis, which can be a potential indicator for developing sepsis, microvascular dysfunction, and multiorgan failure.[68] Myocardial function is depressed in sepsis and is an important prognosticator in the critically ill patients. The degree and recovery time of sepsis-induced cardiac dysfunction is determined by the severity of sepsis, but cardiac dysfunction can be reversible in most survivors. In animal studies, it was found that the amount of transcripts of genes related to the TLR2/MyD88 (TLR2/ myeloid differentiation factor 88) and JAK/STAT (Janus kinase/signal transducer and activator of transcription) inflammatory pathways, β-adrenergic signaling, and intracellular calcium cycling was significantly correlated with the extent of cardiac dysfunction.[97]

As for treatment, there is no specific treatment for sepsis induced myocardial dysfunction. Current general therapeutic strategies include controlling infection, hemodynamic support, modulation of the host response and critical care support.[74] Although a lot of evidence speculates that stress induced myocardial dysfunction as a temporary compensatory mechanism advantageous for patients, but septic shock patients still require the use of inotropic agents and/or vasopressors to maintain adequate mean arterial pressure, oxygen delivery, and myocardial contractility.[60] It also requires an adequate systemic fluid support to maintain perfusion pressures and sufficient blood flow for regional and global demands.

To treat chronic inflammation induced cardiac dysfunction such as the one happening in DMD patients, anitimmune therapy has not been successful yet[98] and cardioprotective therapy is used to ameliorate cardiac remodeling and dysfunction. While efforts continue toward optimizing cardiac and respiratory care for DMD patients, there is no cure for this disease and treatment is limited to glucocorticoids to prolong ambulation and drugs to treat the cardiomyopathy.[98] The treatments used for the DMD cardiomyopathy are based on the weakness of cardiac function. Supportive approaches for ventilation and circulation can be used to improve the quality of life and natural history of DMD patients.[99] Plasma cytokines during chronic inflammation and acute-phase reactants, including TNFα, IL-6, C-reactive protein, and fibrinogen, can be monitored in DMD to follow-up the progression of the disease.[100]

### Cardioprotective therapy

As aforementioned, despite treating the primary diseases, cardioprotective therapies for acute or chronic inflammation induced myocardial dysfunction are important. In sepsis or DMD patients, β-blockers as a classic drug to treat cardiac diseases are not only having the effects of preventing ischemia, decreasing oxygen demand, and TNF production[7]; they also reduce local and systemic inflammation.[101] Cardiac inotropy can be increased by levosimendan, istaroxime, or omecamtiv mecarbil without greatly increasing cellular oxygen demands. Heart rate reduction with ivabradine reduces myocardial oxygen expenditure and ameliorates diastolic filling. Immunomodulation and nutrition support are also important to protect cardiac function because stress and inflammation are catabolic processes. All these treatments can be used preventively.

### β-adrenergic blockade and angiotensin II receptor blockade

The benefits of β-blockade have been demonstrated in clinical trials of pediatric severe burn, heart failure, severe trauma, and traumatic brain injury.[7] As β-adrenergic stress is a major factor in sepsis-induced myocardial dysfunction, the use of β-blocking agents could be beneficial. However, this is still controversial as it may have potential negative inotropic effect in patients with sepsis induced myocardial dysfunction. There are several experimental studies using β-blockers in sepsis, showing mortality reduction if commenced before a septic insult.[7] In an animal model with sepsis, β-blocker showed promising effect on reducing heart rate, maintaining cardiac stroke volume, and reducing inflammation and the organ injury.[102–104] In burned children, treatment with propranolol (a nonselective β-blocker) during hospitalization attenuates hypermetabolism and reverses muscle-protein catabolism.[105] Preexisting β-blockers treatment has the potential to improve adult burn outcomes as well.[106] However, post injury treatment needs to be further studied in randomized clinical trials too. Study with two rat models, adjuvant arthritis and subcutaneous air pouch edema, showed antiarthritic effects by Carvedilol. Carvedilol, a α1-, β1-, and β2-adrenergic receptor blocker that can increase eNOS activity,[70] attenuated leukocyte migration, oxidative stress response, and the production of proinflammatory cytokines (TNF-α and IL-6) and eicosanoids (PGE2 and LTB4).[107] Besides traditional inhibition of AT1 to AT2 receptors, angiotensinogen converting enzyme (ACE) inhibitors also have positive effects on chronic inflammation in atherosclerosis. Though they cannot reverse cardiac remodeling, they can reduce acute cardiovascular events during inflammation.[108,110] Angiotensin receptor blockers (ARBs) have the similar clinical effects as ACE inhibitors though they work through different mechanisms, like increasing NO bioavailability and improving insulin sensitivity.[70]

### Levosimendan

Levosimendan is a new pharmacological agent used in the management and treatment of acute and chronic heart failure. Unlike other inotropic agents, as a myofilament calcium sensitizer, it can improve cardiac performance without activating the SNS and changing oxygen consumption. Levosimendan binds to the N-terminal of troponin C with high affinity at high [Ca^2+^]_i_ that can only be reached during systole, prolonging the interaction of myosin and actin filaments through the inhibition of troponin I.[111] Levosimendan have been applied in some observational studies, such as a perioperative use, cardiogenic shock, sepsis, and right ventricular dysfunction.[112] Levosimendan has vasodilatory and cardioprotective effects mediated by opening ATP-dependent K^+^ channels and mitochondrial ATP-sensitive potassium (mito.K(ATP)) channels in cardiac myocytes and vascular smooth muscle cells.[113] This inotropic agent also has mild PDE inhibitory action.[114] Moreover, there is evidence that levosimendan has additional anti-inflammatory effect in the prevention or treatment of acute or chronic inflammation depending on the pathogenic factor.[115] Doorduin and coworkers[116] found levosimendans improve contractile function of the diaphragm in the acquired diaphragm muscle weakness patients. However, there is no related report using levosimendan to treat DMD that can potentially be treated with this drug.

### Ivabradine

Ivabradine is a selective inhibitor of the hyperpolarization activated cyclic-nucleotide-gated I_f_. I_f_ is important for generating the pacemaking current, and responding to β-adrenergic stimulation of the SAN cells. The (I_f_) blocker ivabradine reduces heart rate and improves systolic function without causing apparent direct hemodynamic effects. Contrary to β-blockers, ivabradine has no hypotensive character. When body suffers stress including sepsis, burn, and trauma; the heart rate is accelerated causing tachycardia.[117,118] Lots of studies have demonstrated that the heart rate is a major independent cardiovascular risk factor for adverse prognosis.[119] Reduction of heart rate is the main effect of ivabradine and protects the myocardium against ventricular dysfunction induced by tachycardia. These effects can improve endothelial function, modulate immune cells and enhance a long-term adaptation in calcium handling.[120] In a dystrophy case report, ivabradine normalizes sinus tachycardia and heart failure in Becker’s muscular dystrophy patients with dilated cardiomyopathy and reduces arrhythmic events and left ventricular remodeling.[121]

### Prospect

Both acute (e.g., sepsis and acute myocarditis) and chronic inflammation (e.g., muscular dystrophy) can cause cardiac dysfunction, which is an often cause of mortality and morbidity in medical conditions causing inflammation. However, inflammation may also exert protection on the heart. Many mechanisms including signaling pathways involving cytokines and chemokines, NO, and Ca^2+^ and β-adrenergic system are underlying both the detrimental and salutary effects of inflammation. Anti-inflammatory clinical trials have not succeeded thus far. Current treatments for cardiac dysfunction after inflammation focus on cardiovascular support, limiting cardiac injury and adverse remodeling with inhibition of b-adrenergic and the reninangiotensin-aldosterone system (RAAS) systems, and reducing heart rate. A better understanding of the underlying mechanisms for both detrimental and protective effects of inflammation under different conditions in the heart is needed for future development of effective therapy to prevent cardiac injury caused by inflammation while at the same time to preserve necessary and protective aspects of inflammation.
